# Moving towards a new vision: implementation of a public health policy intervention

**DOI:** 10.1186/s12889-016-3056-3

**Published:** 2016-05-17

**Authors:** Ruta Valaitis, Marjorie MacDonald, Anita Kothari, Linda O’Mara, Sandra Regan, John Garcia, Nancy Murray, Heather Manson, Nancy Peroff-Johnston, Gayle Bursey, Jennifer Boyko

**Affiliations:** Faculty of Health Sciences, School of Nursing, McMaster University, 1280 Main Street West, Hamilton, L9S 4K1 ON Canada; School of Nursing, University of Victoria, 3800 Finnerty Road, Victoria, BC V8P 5C2 Canada; School of Health Studies, University of Western Ontario, Arthur & Sonia Labatt Health Sciences Building Room 222, 1151 Richmond Street, London, ON N6A 3K7 Canada; School of Health Studies, University of Western Ontario, Arthur & Sonia Labatt Health Sciences Building Room 403, 1151 Richmond Street, London, ON N6A 3K7 Canada; School of Public Health and Health Systems, Faculty of Applied Health Sciences, University of Waterloo, BC Matthews Hall, Room 2310, 200 University Avenue West, Waterloo, ON N2L 3G1 Canada; Public Health Ontario, 480 University Avenue, Suite 300, Toronto, M5G1V2 ON Canada; (Formerly with the) Public Health Standards, Practice and Accountability Branch, Population and Public Health Division, Ontario Ministry of Health and Long-Term Care, 393 University Ave. Suite 2100, Toronto, ON M7A 2S1 Canada; Region of Peel, 10 Peel Centre Drive, Brampton, L6T 4B9 ON Canada

**Keywords:** Public health, Policy, Health policy, Implementation, Implementation science, Knowledge translation

## Abstract

**Background:**

Public health systems in Canada have undergone significant policy renewal over the last decade in response to threats to the public’s health, such as severe acute respiratory syndrome. There is limited research on how public health policies have been implemented or what has influenced their implementation. This paper explores policy implementation in two exemplar public health programs -chronic disease prevention and sexually-transmitted infection prevention - in Ontario, Canada. It examines public health service providers’, managers’ and senior managements’ perspectives on the process of implementation of the Ontario Public Health Standards 2008 and factors influencing implementation.

**Methods:**

Public health staff from six health units representing rural, remote, large and small urban settings were included. We conducted 21 focus groups and 18 interviews between 2010 (manager and staff focus groups) and 2011 (senior management interviews) involving 133 participants. Research assistants coded transcripts and researchers reviewed these; the research team discussed and resolved discrepancies. To facilitate a breadth of perspectives, several team members helped interpret the findings. An integrated knowledge translation approach was used, reflected by the inclusion of academics as well as decision-makers on the team and as co-authors.

**Results:**

Front line service providers often were unaware of the new policies but managers and senior management incorporated them in operational and program planning. Some participants were involved in policy development or provided feedback prior to their launch. Implementation was influenced by many factors that aligned with Greenhalgh and colleagues’ empirically-based Diffusion of Innovations in Service Organizations Framework. Factors and related components that were most clearly linked to the OPHS policy implementation were: *attributes of the innovation itself*; *adoption by individuals; diffusion and dissemination;the outer context – interorganizational networks and collaboration; the inner setting – implementation processes and routinization; and, linkage at the design and implementation stage.*

**Conclusions:**

Multiple factors influenced public health policy implementation. Results provide empirical support for components of Greenhalgh et al’s framework and suggest two additional components – the role of external organizational collaborations and partnerships as well as planning processes in influencing implementation. These are important to consider by government and public health organizations when promoting new or revised public health policies as they evolve over time. A successful policy implementation process in Ontario has helped to move public health towards the new vision.

**Electronic supplementary material:**

The online version of this article (doi:10.1186/s12889-016-3056-3) contains supplementary material, which is available to authorized users.

## Background

Public health systems in Canada have undergone significant renewal over the last decade in response to various threats to the public’s health such as severe acute respiratory syndrome (SARS) [1, 2], water contamination [3], and Hepatitis C in the blood supply [4]. In Ontario (ON), Canada, renewal efforts have involved the review and subsequent revision of public health policies that “establish the minimum requirements for fundamental public health programs and services…” to be delivered by ON’s 36 boards of health [5]. There is limited research on how these policies have been implemented or what has influenced their implementation. Understanding the process of policy implementation in practice settings can provide valuable learning for other jurisdictions undergoing similar public health policy renewal processes, and is the focus of this paper.

The aim of Renewing Public Health Systems (RePHS) – a five year program of research – is to study the implementation and impact of public health renewal in two Canadian provinces, ON and British Columbia (BC), over time. Two exemplar programs – chronic disease prevention (CDP) and sexually-transmitted infection prevention (STIP) – are being used to explore these topics. These programs were chosen based on having a similar focus in both provinces allowing for provincial comparisons, the likelihood of primary care and public health collaboration on the topic (a secondary research interest) and expressed interest by our knowledge user partners. This paper reports on results from the first two years of data collection (baseline) in 2010–11 in ON. Baseline results from BC are reported in a parallel paper [6].

### OPHS policy development

Five years after the 2003 SARS outbreak, the Ontario Public Health Standards 2008 (OPHS) [5] were released. Additional file 1 provides a detailed history of the process of initiation, development, and roll out of the policies up to 2010 when this program of research was initiated. In short, following the 2003 SARS outbreak [7, 8] a provincial report – *Revitalizing Ontario’s Public Health Capacity* [9] – recommended that the Mandatory Health Programs and Services (MHPSG) [10] policy document be replaced with renewed provincial public health program standards that were to be continually revised, in other words, part of an ever-greening process. The Standards were expected to fit within public health’s fiscal envelope or public health funding. In addition, there was to be increased emphasis on accountability, coordination and collaboration across the health system, as well as financial sustainability. Government and a Technical Review Committee deliberated on a number of cross-cutting themes to be included in the new Standards [11]. These included: balancing the need for provincial standardization and being responsive to local contexts; integrating public health functions as part of the overall framework emphasizing population health assessment and surveillance, as well as the delineation of a Foundational Standard; establishing a logic model approach to define causal linkages between requirements, board of health and societal outcomes; and, ensuring that public health roles and contributions to addressing determinants of health and reducing health inequities was prominent within a robust introduction to the standards.

**Fig. 1:**
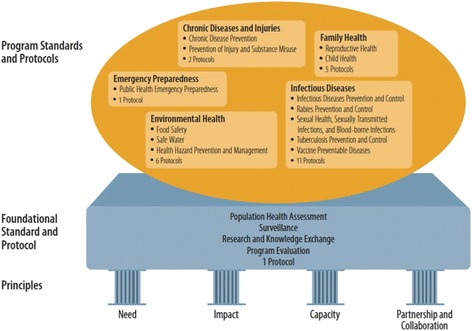
Ontario Public Health Standards 2008. The Ontario Public Health Standards [1] framework is supported by four pillars which are the principles that underpin the Standards. The foundational Standard and protocol is to be integrated into the 5 program Standards with their relevant accompanying protocols. This diagram was current at the time of data collection: In May 2014, it was revised and an additional protocol was added.

The draft OPHS were submitted to the Chief Medical Officer of Health in April, 2007 with an expectation that “a robust and comprehensive roll-out strategy” would be included with training supports. On the Technical Review Committee’s advice, government continued to develop protocols to support specific requirements within the OPHS to achieve greater standardization in province-wide implementation. Figure 1 illustrates the four pillars (or principles), upon which the Standards were built, the OPHS Foundational Standard [5] and its accompanying Population Health Assessment and Surveillance protocol [12], as well as five program standards and their relevant protocols that existed at the time this study was conducted. This figure was updated in May 2014 to reflect the addition of the *Tanning Beds Compliance Protocol* [13].

In 2007, Protocol Development Teams began drafting protocols, such as a Population Health Assessment and Surveillance Protocol and Infectious Diseases protocol [12, 14]. Numerous stakeholders had an opportunity to comment on drafts in 2008. Guidance documents were also developed, updated or referenced. The OPHS and 26 protocols were released in October 2008 and came into effect in January 2009 and continue to be updated, with the most recent revisions to the 2008 Standards published in 2014 [15] with an additional protocol.

The OPHS release in 2009 included program specific workshops and a dedicated website for the OPHS with additional resources. In support of the OPHS release, the former Ministry of Health Promotion and Sport released nine guidance documents in 2010. The content of these policy documents has been explored in depth by the RePHS research team from a number of perspectives including: chronic disease prevention [16], equity [17], public health human resources [18], and primary care and public health collaboration [19].

### An integrative framework to understand implementation of a public health policy intervention

A focus on implementation sciences grew in parallel with the development of the field of knowledge translation, but this focus was on the knowledge user setting and understanding how a knowledge product or innovation could be integrated in everyday practice or policymaking in a sustainable way. As a result, attention to how contextual and organizational factors enabled implementation became important [20, 21]. Greenhalgh and colleagues wrote a seminal article and book that reported on a systematic review to address how innovations can be spread and sustained in health services organizations [22, 23]. They used a meta-narrative technique to synthesize findings from empirical and theoretical works in 13 different research traditions, such as evidenced-based medicine and complexity studies. Based on their analysis, the authors put forth a “unifying conceptual model” of diffusion in service organizations composed of eight elements: 1) *the innovation*, 2) *adoption by individuals*, 3) *assimilation by the system*, 4) *diffusion and dissemination*, 5) *system antecedents for innovation*, 6) *system readiness for innovation*, 7) the outer context: *interorganizational networks and collaboration*, 8) *implementation and routinization, and 9) linkage (at the design and implementation stage)*. We used this empirically-based framework [22, 23], to interpret our data related to the policy implementation. In our analysis we took the authors’ advice also to pay attention to implementation adaptations that arose and occurred informally in addition to those that were spread through official, planned mechanisms.

The purpose of this paper is to explore public health service providers’, managers’ and senior managements’ perspectives in six Ontario health units on the process of implementation of the OPHS and factors influencing their implementation within two exemplar public health programs – CDP and STIP. We used Greenhalgh et al’s *Model of Diffusion of Innovations in Service Organizations* as an organizing framework [22, 23] to interpret the findings. Similar to the parallel paper that explored policy implementation of BC’s Core Public Health Functions Framework, “knowledge about the implementation experience and the challenges encountered by practitioners and managers may help inform improvements in both the policy intervention and the implementation process” [6] p.4.

## Methods

This paper reports on baseline results from a longitudinal multiple case study, which incorporated a number of data collection methods. We drew upon results from focus groups and interviews to obtain perspectives from health unit personnel and senior management in relation to the core program standards. Cases were defined as the STIP and CDP programs that are directed by policy. These programs were chosen because they were offered in both provinces and were likely to involve collaboration with primary care - a cross cutting theme. Six out of 36 health units in the province of Ontario were recruited to provide a diverse sample that matched the general demographic makeup (e.g., population density; ratio of immigrants; urban, rural, remote or mixed) of the health authorities in BC. One health unit dropped out due to reduced capacity to participate in research as a result of multiple staff changes and vacancies; the health unit was replaced by another in 2011. Service providers, managers and senior management (i.e., directors, a Chief Nursing Officer, Medical Officers of Health, and Associate Medical Officers of Health) were recruited to participate (Table 1). Service providers and managers participated in focus groups separately to avoid the influence of power-over relationships. Focus groups were conducted within homogenous teams (i.e., STIP and CDP) when relevant. Senior management participated in interviews. Interviews and focus groups were audio-taped, transcribed verbatim, cleaned for accuracy and entered into NVivo10.0 for analysis. Focus groups were conducted face-to-face at the health units by program area, and interviews were completed in person or by phone by researchers. Focus group and interview questions were tailored to address participant groups, i.e., service providers, managers, senior management (See Additional file 2).

**Table 1:** Number of Participants by Type, Data Collection Strategy and Program (2010–2011)

Participant Type	Focus groups (# of FGs)		Interviews			Total
	CDP	STIP	CDP	STIP	Mixed Programs	
Service providers	40 (6 FGs)	34 (6 FGs)	-	-	-	74
Managers	21 (4 FGs)	20 (5 FGs)	3	1	-	45
Senior managers	-	-	-	-	14	14
Total ON	61	54	3	1	14	133

In this study, we used concepts in Greenhalgh’s framework as sensitizing concepts to provide general ideas for developing our interview questions to ensure that we covered the broad domain of known influences on successful policy and innovation implementation. When it came time for analysis, we did not use Greenhalgh’s framework to structure or organize our coding. Rather, we coded line by line in inductive fashion to develop a set of empirically-derived codes. To begin, a coding framework was created by having each researcher from the team code two or three selected transcripts followed by a review and discussion in a face-to-face meeting. Agreement was reached on the coding framework and remaining transcripts were coded by trained staff. After a few more transcripts were coded, results were reorganized by identifying codes that could be merged or reorganized into higher level thematic codes by researchers. When all coding was completed, each researcher was assigned a set of transcripts to review as a peer debriefing strategy: in other words, each transcript was reviewed by two researchers to increase the credibility of analysis [24, 25].

In the final step, after thematic higher order codes were identified, we again turned to Greenhalgh’s framework to help us in interpreting the various categories of inductive codes and the relationships among them. Our discussion is therefore framed around relevant concepts in Greenhalgh’s framework. Nonetheless, we acknowledge that in our coding we were theoretically sensitized by the Greenhalgh framework. Sensitizing concepts are central to a grounded theory approach to analysis [26], but the notion was developed much earlier by Blumer [27]. A sensitizing concept is one that is not clearly specified with respect to its attributes. Thus, it does not allow the researcher to move easily between data identifying the instance and the attributes of the concept. Rather, a sensitizing concept provides the researcher with general suggestions about what to look for in an empirical instance. Definitive concepts, on the other hand, provide very specific directions for what to see [27] p. 7.

Key results were circulated to decision-maker team members (they were not privy to the raw data) as well as senior Ministry staff at various meetings for input. Thus decision-makers provided additional context to help interpret findings and increase credibility of results [28]. An integrated knowledge translation approach [29] was used throughout, and is reflected by the inclusion of academics as well as decision-maker researchers (i.e., government and public health unit staff) on the RePHS team.

### Ethics and consent

Ethics Board approvals were obtained from McMaster University Hamilton Integrated Research Ethics Board (HIREB); Public Health Ontario through the University of Toronto (Office of Research Ethics); Ottawa Public Health, Ottawa Public Health Research Ethics Board; Sudbury & District Health Unit, Sudbury & District Health Unit, Research Ethics Review Committee (RERC); and Toronto Public Health, Toronto Public Health Research Ethics Board. All participants consented to this research.

## Results

Participant demographics will be presented followed by results, using illustrative participant quotes, related to: 1) how the OPHS (policies) were disseminated; 2) awareness of policies; 3) general opinions about the policies; 4) processes used for policy implementation; and 5) internal and external factors influencing policy implementation.

### Participant demographics

**Fig. 2:**
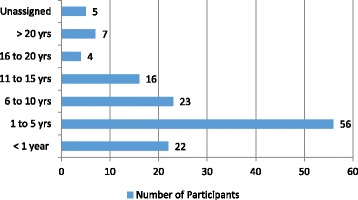
Number of Participants by Years in Position

A total of 21 focus groups and 18 interviews were conducted throughout October-December 2010 (manager and staff focus groups) and August 2011 (senior management interviews) (Table 1). A total of 133 individuals participated from six participating health units. Of all participants, there were 74 (55.6 %) nurses; 17 (12.8 %) health promoters; 13 (9.8 %) dietitians; 5 (3.8 %) nutritionists; 5 (4.8 %) physicians; 4 (3.0 %) social workers; 1 (0.8 %) community worker; and 9 (6.8 %) ‘other’ [missing data n = 5 (3.8 %)]. Of all participants: 22 (16.5 %) worked for under a year in their current positions; 56 (42.1 %) for 1–5 years; 23 (17.3 %) for 6–10 years; 16 (12.0 %) for 11–15 years; and the remaining 11 (8.3 %) were in their positions for over 15 years [missing data: n = 5 (3.8)] (see Fig. 2).

### Policy dissemination at the health unit level

Policies were most frequently communicated through formal means by health unit managers to front line service providers. This was done through program discussions, new staff orientation, presentations, in-services, emails and workshops. In some health units, managers noted that communication “cascaded” from senior management to service providers and policy analysts, and in one health unit a designated ‘champion’ was responsible to ensure policies were communicated through programs/branches (satellite offices). Sometimes participants were informed about the policies by external sources such as: external networks, Ministry of Health and Long-Term Care updates, Ministry sponsored webinars, and the Ministry’s formal launch. A director noted: “I think mostly it was in the initial consultations. So there was that launch of the drafts and all the health units were asked to provide feedback.” Often managers and front line staff were involved in standards development by providing feedback, or contributing to the writing process. This helped to incorporate a variety of perspectives. As described by a service provider:

… I met with the tobacco cessation people [at the Ministry] as they rewrote those mandatory guidelines […]. And from my perspective what was needed as far as cessation goes in the various communities throughout Northern Ontario, I guess that’s what we were representing.

In a few cases policies were reviewed by staff independently, staff assisted in the preparation of guidance documents, or they participated on a provincial planning committee.

### Awareness of the new policies

Managers and senior management knew about the new policies and reported that staff awareness was supported through discussions at team meetings or regular planning meetings. As described by one manager:

We were aware of them and used them as a planning tool before, even before they were passed. So, I think we were very aware of them and we try to really encourage our staff to continue to be aware of them.

In a few health units the OPHS were incorporated into organizational documents, such as strategic plans and logic models, as a way to identify and align priority program activities with the government mandate thereby facilitating policy implementation into services and programs

Service providers were asked about their knowledge of the policies. Many were unaware of them, or were only aware of some aspects of them, such as the accompanying protocols. As noted by one participant: “I’m familiar with the OPHS but the old ones (referring to MHPSG). I haven’t seen the new [OPHS], updated ones. They have not been, in any way that I’m aware of, been shared with me.” For most front line staff who were aware, they did not focus on them “… I don’t think about the standards an awful lot. Like in the clinic here, you are focused on hands-on work. So it’s not something that we dwell on too much. We know what we have to do and we do it.” Despite direct involvement by health unit staff in the development of the OPHS and broad health unit communications around them, awareness was uneven. Overall, managers were more aware and found ways to incorporate them into their work processes, whereas most front line service providers either were unaware or paid little attention to the OPHS. Having more direct engagement with the policies through the development and or implementation phases seems to have influenced the level of awareness of the policy.

### Influences on policy implementation

**Fig. 3:**
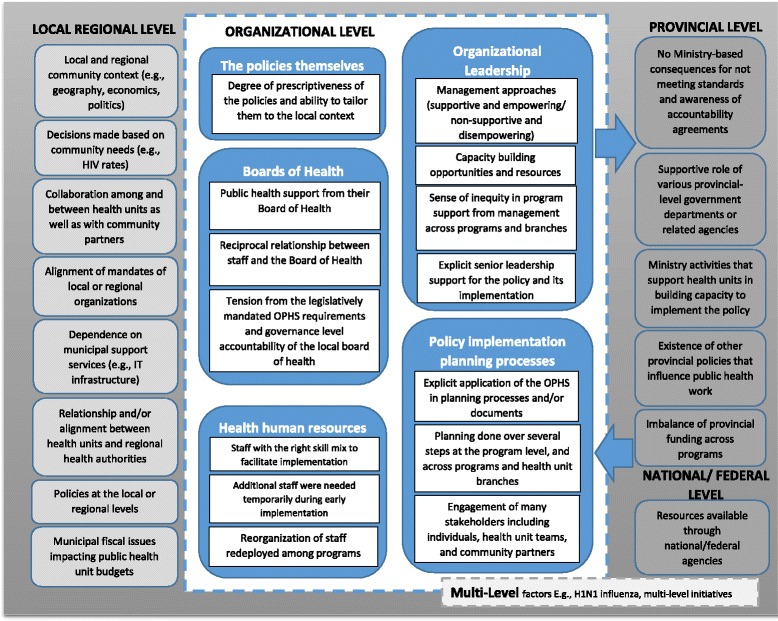
Factors influencing policy implementation

Themes that indicate influences on policy implementation are **bolded** and summarized in Fig. 3.

#### *The OPHS themselves:* general opinions about policies

There were mixed views about the OPHS. In general, some managers and senior management reported that they were fortunate to have them, describing them as providing direction and being empowering. As a director noted: “… it does provide direction for us. It is a way that we have structured our organization somewhat around the Standards. It’s certainly how we report out on things.” Others saw the policies as being similar to the old policies with the same overarching themes or as an extension of the MHPSG. A few others understood them as continually emerging and changing, with their new protocols and guidance documents.

There was a mix of opinions around the **degree of prescriptiveness** of the OPHS compared to the MHPSG. However, most participants felt the OPHS were generally less prescriptive allowing for more flexibility in programming, but providing less direction. Exceptions were related to aspects of infectious disease programs which were seen as more prescriptive. As a STIP manager described: “It’s not prescriptive as far as the education piece, but the clinical and the investigation, that’s very prescriptive.” There was a need for balance between having flexibility in implementing the OPHS while also being prescriptive so that arguments could be made for some hard to sell programs, such as harm reduction. A manager explained:

The lack of information [referring to the non-prescriptive policy] allows us to do what we’d like to do. However, that same lack of information also doesn’t give us the permission that we need to do some of the things that we need to do. So, would we have liked to have seen more meat? Maybe.

Participants indicated that the non-prescriptive nature of the policies left decision-making around them open to outside influences, which will be described later. Some tension was identified in that the policies called for evidence-based programming. However, participants felt that there were times when specific, local community needs were not prioritized areas in the OPHS or no evidence existed in relation to interventions to address community needs. Participants also correctly understood that the policies were to be fiscally neutral – in other words, staying within the budget. This limitation was generally accepted by participants as a reality that could not be changed.

#### Organizational leadership

Organizational leadership was a significant factor influencing policy implementation. The topic of leadership was spoken about as both a barrier and facilitator by staff and managers from most health units. A significant factor influencing implementation was **management approaches**. Some staff in a number of health units said they felt unsupported and disempowered. Complaints ranged from challenges to autonomous practice of service providers, increased control by senior management, a lack of staff voice, lack of transparency related to changes in priorities, limits on innovation, and having to work through multiple layers of bureaucracy. One staff member described this latter issue as follows: “…they have added layers and layers and layers. I used to report to a manager; now I have a supervisor and I have a manager. There’s the MOH.” Another staff member described their lack of control:

You are suddenly doing something that just changes your […] entire operational plan and allocation of resources. And we are often not given a reason. It could be someone’s personal interest: It could be something they heard on the news. But it just plops into your assignment and we don’t have a lot of say at that level.

On the other hand, leadership was seen by many others as being trusting, supportive, flexible, accessible, and encouraging creativity. As one staff member put it: “I met with the supervisor of the population health team and we talked about the scope of this position as well as the standards and planning for this upcoming year. So she was very supportive and just assisting me with that planning.”

**Capacity building opportunities and resources** was another important factor influencing implementation of the policies. Staff in most health units expressed the presence of and value in capacity or career building opportunities, as well as access to supportive tools and resources; however, not all staff members shared this sentiment. One participant said: “I don’t feel supported in terms of responsibilities with the new policies. They tell us what we are supposed to do but I don’t think the process and how we do it is well supported…”

Another leadership factor influencing implementation that was mentioned in most health units was a sense of **inequity in program support from management across programs and branches**. In particular there was less support for some topics over others. Topics that did not explicitly fall under the policies were less valued although they were important to communities, for example: “body image: there wasn’t quite a good fit under OPHS […] Is that mental health? Because, if it’s mental health, then it doesn’t belong in public health. If it’s healthy eating, then sure.” Also, health promotion was less valued by managers than infectious disease control; and sensitive topics such as harm reduction were valued less than topics such as healthy weights.

**Explicit senior leadership support for the policy and its implementation** was reported to be important by all. Having a vacant senior leadership position at the time of the roll out was a barrier for implementation in some health units, while other health units were influenced by local municipal pressures. A lack of valuing of OPHS by leadership and lack of leadership to champion the OPHS were problematic in half of the health units. As shared by one participant: “The lack of importance placed on [the policies], from the top down impacted how they were received by staff.” Another senior manager supported perceptions from some health units that: “We could have used a little bit more leadership and guidance in terms of how we can do these things and what are some of the resources.” Medical Officers of Health were identified by some health units to have a very important influence on public health decisions in relation to the roll out of policies and accompanying program resources. For example, a manager described a situation in which the Medical Officer of Health did not support the provision of services to homeless populations as they were seen as peripheral to STIP programming: “…It kind of brought to light maybe things that we were doing that didn’t exactly fit in with our program or didn’t fit in with the role of public health.” In this case, it showed the power that leaders had in interpreting the policy and ultimately making decisions about program resources. Although some staff said it was up to leadership to deal with implementation, others said they wanted more involvement in it. This has the potential of creating staff discontent from a feeling of disempowerment.

#### Policy implementation planning processes

Health units reported on the **explicit application of the OPHS in planning processes and/or documents**. The policies were used by HUs most often to justify programs as well as to guide operational planning and other program planning processes (balanced scorecards and the budgeting process). This was in addition to the application of various forms of evidence to inform planning (e.g., literature reviews, environmental scans, other health unit activities). Formal planning tools and planning processes were generally used (e.g., logic models, work plans, service plans, and internal planning processes). A number of participants reported making explicit reference to the OPHS in planning documents. One director explained how the relationship between the OPHS and financial resources was explicitly noted in planning documents: “We’ll show what percentage [of our budget] is going to Foundational Standard or different Standards.” OPHS implementation was facilitated by the explicit integration of the policy in program planning processes including decision making around the use of resources.

Participants described planning processes as involving **several steps at the program level, as well as across programs and health unit branches**. A manager described the process this way.

So we have to develop logic models for each of our program areas, pretty well – particularly at the standard level and then at the program level. And then we have to develop yearly program plans […] Those are written by the managers and staff and reviewed and approved by the director then onto the medical officer of health. And of course, those are compared to the standards and protocols to ensure that we are meeting all of the requirements.

Others said that integration across programs and branches in planning processes could be improved: “I think there could be more integration across the branches. Currently we have good integration within our [CDP] branch […] We try very much to work with healthy sexuality program…” Also, planning processes appeared to be more extensive for CDP than STIP. A STIP manager explained:

… We are different, because we have very protocol-driven programs. We really don’t have a lot of choice in this. We have a choice in how we target the high risk. So there’s room to move, but it’s not the same as chronic disease where you’re planning new programs all the time.

Planning processes **engaged many stakeholders including individuals, health unit teams, and community partners**. Planning that incorporated staff and management feedback and involved the full team was identified as vital in the planning process. As depicted by a STIP manager: “…in the planning process when we do our operational plans… it would be with our front-line staff. And the staff that’s going to be doing the clinical or the education portion. So [we plan] as a team.” Participants also spoke at length about the role communities played in planning programs and services to implement the OPHS. Much of planning was community driven, community engaged, as well as community needs driven. Communities were engaged through community consultations, surveys, assessments, focus groups, or advisory committees. A CDP manager noted:

We have placed more emphasis on the need, in terms of gathering evidence, to linking with priority populations and engaging with communities […] Some of the things that we have decided to do with respect to our four branch office areas in particular is have community consultation sessions within [the] branch office areas.

Planning with community partners was also seen as challenging considering the time needed to build trust, respond, and allow them “to be part of that plan as opposed to rushing it.” Despite the time and resources required, engagement by internal staff and external partners in planning processes was essential in implementing the OPHS.

#### Boards of health

Elements of governance by the Boards, which serve to establish norms and structures through management practices, polices and processes, had an influence on the implementation of the OPHS. Many respondents’ comments reflected a perception that **public health was generally well supported by their Board of Health**. In reference to the OPHS, a manager explained that: “… [The Board members] believe in public health. They believe in us. They have confidence in the workers in public health”, implying that trust in public health work extended to the implementation of new practices. This was reflected in a true desire to see positive community outcomes, to be “making a difference in the population’s health.” Further, there was evidence of a **reciprocal relationship between staff and the Board**; as a senior manager said, “I’m really happy with […] the quality of questions and inquiry and interest in public health. The majority of the board members seem to come with that real interest and engagement…” This generally positive interaction led to “pretty high standards” or expectations from public health staff with respect to OPHS implementation. In turn, Boards had the ability to raise the profile of PH by putting PH issues on the political agenda in various cities and regions which was an unexpected, immediate impact of implementation.

In a few cases, however, health units experienced difficulty in attracting the attention of the Board because of the multiplicity of local issues on the table. Further, approvals from Boards of Health were generally required for new programming: “We can’t just say, we’re going to have a new sexual health program, and we’re going to hire new staff […] We have to justify increases in budget, increases in staffing…” This quote hinted at an underlying **tension from the legislatively mandated OPHS requirements and governance level accountability of the local board of health**. Directions from the province and political pressures from Boards of Health who “held the purse strings”, and thus make decisions around programming, were not always aligned creating challenges for OPHS implementation.

#### Health Human Resources

Public health human resources played an important role in supporting policy implementation. Front line staff spoke to the importance of additional resources to assist with implementation of OPHS such as additional staff and **staff with the right skill mix to facilitate implementation** of revised or new programs and services. For example, increased emphasis on the use of evidence and research required staff with competencies to support this. One participant indicated: “I think our role did not change but the support that we were providing to teams in terms of consultation for evaluation and things that did increase a little bit more”. In addition, participants identified that **additional staff were needed on a temporary basis during early implementation** or new roles were created as a result of needing to meet OPHS requirements and the Foundational Standard in particular [5]. Participants from some HUs indicated that additional resources, particularly additional human resources, were needed to support implementation of OPHS but were not obtained.

In some HUs, a reorganization of programs and services to better implement OPHS led to **staff being redeployed among programs**. This meant that some staff moved from programs where they had worked for many years. Some staff were unhappy with these changes to their role. Others commented that staff were working across teams which was creating better collaboration and sharing of resources in their health units. Some participants described how the OPHS brought greater clarity to their role by focusing planning on key public health programs and services to be delivered.

…it’s forcing us to think about, ‘okay, what are the policy implications’? And now I’m hearing staff understand what that might look like. They have a vision of where that might lead particularly in the chronic disease, injury prevention area.

Many front line staff suggested that the OPHS reflected the reality of public health practice and programs. They indicated that they were already meeting the OPHS in their day-to-day work and OPHS implementation had little impact on their roles:

For me it never changed, you know, for me it was always kind of like, we need to get out there…So I had no idea if that was our goals or whatever. But at the end of the day, you know, I knew that we were doing it. And I think that we all knew that we were doing a good job.

#### Local/Regional factors

The **local and regional community context**, including population make up, geography, economics, and politics, had a significant influence on policy implementation. Whether a health unit served a rural population, a vastly diverse immigrant population, or offered public health services near an international airport, these contexts influenced the delivery of programs and services. As a senior manager explained, rural access “has an impact on our delivery [and] the cost of delivery of services, because of travelling and having people in the smaller areas and the areas where there’s need. We need to be looking at that, which the Standards don’t.” As noted earlier, the local political climate also influenced OPHS implementation. For example, access to contraband cigarettes was described as a barrier to implementation of a protocol in a health unit surrounded by tobacco farms. Many examples were provided about smoking prevention efforts being influenced by local politics, particularly where the community’s economic base was tied to tobacco:

…specific to chronic disease, we have an agricultural community growing tobacco and having managed that program for many years, that’s a real political challenge. Moving forward and trying to do the programs as best we can, but knowing that they’re often not supported strongly by the politicians, or being lobbied by their farming community. And it creates a tension there within decision making.

A senior manager pointed out that **decisions were made based on community needs**. Another senior manager supported the view that community data matters: “…given our high rates of HIV and the issues around drug use and harm reduction […] we’re working with particular populations. We have a high new immigrant population […] We’re focusing our effort.” Another key factor was the needs of local communities and organizations. As one STIP manager described, “There’s a fair bit of flexibility in some of these standards for local needs to be driving some of it.” Community need was sometimes trumped by research evidence creating a dilemma for staff:

You want to focus on a certain area and there isn’t enough say best practices or evidence to support it, I think there’s less support to do that. It’s more, this is concrete, there’s proof behind it, you work on that… and sorry about the community wanting to work on this, but there’s not enough evidence behind it.”

Front line staff in particular struggled with this, since it threatened long-term relationships with their community partners.

A key implementation facilitator was **collaboration among health units and between health units and community partners**. Collaborations enhanced community partners’ awareness of public health services, as well as implementation of school, street outreach, and primary care programs. Collaboration also enhanced access to specific communities (e.g., cultural, geographic, hard to reach communities). As one STIP manager put it.

…street outreach […] has all been done through collaboration with partner agencies. Because when we do street outreach, we are actually walking the streets and encouraging people to come in for testing in conjunction with the Needle Exchange Program and the outreach workers there or Access AIDS and their outreach workers.

Another factor supporting implementation was **alignment of mandates of local or regional organizations**. Collaboration is a two way street with NGOs looking to health units for support in implementing their work, and health units looking to these same groups to implement their guidelines. For example, regional cancer programs and public health units collaborate to implement aspects of Ontario’s Cancer Plan related to healthy eating and physical activity. Although most participants saw community partners as resources to support program delivery and evaluation, others saw them as competitors for limited resources. Interestingly, a few saw partners as drivers for PH implementation. As one manager explained: “[Partners] are trying to leverage [resources]. [They tell you] ‘your mandate says you should be doing this.’”

**Dependence on municipal support services** was another factor influencing implementation and was usually reported as a barrier*.* For example*,* reliance on municipal infrastructure for IT systems to support surveillance work. As noted by a senior manager: “Where we want support of an IT department, we have to negotiate that with the City*.”* Another example of reliance on municipal structures was the use human resource departments, requiring health units to negotiate with them for services.

Another regional factor was the **relationship and/or alignment between health units and regional health authorities**. Funding flows to all health services from the regional health authorities with the exception of primary care and public health who generally receive their funding from the province. One public health manager described the regional authority’s role and their relationship to it as follows:

Their mindset is not chronic [disease prevention-oriented] and their mindset is not…primary, secondary, tertiary, and even quaternary care… and public health is rarely [their interest]. I was invited to [a committee] which was basically… physician advisory groups… [We] sat and decided [what] the clinical plan was going to be. But it’s clear from that clinical plan that public health is just a bump on a log and they’re not really interested because they don’t fund us.

Other managers spoke about positive involvement with the regional health authority for regional program development such as falls prevention and cardiovascular disease prevention, as well as benefits of linking with primary care through various regional committees.

**Specific policies at the local or regional levels** also affected implementation of the standards. For example, at the time of OPHS implementation, there was a regional focus on performance measurement in one health unit which diverted energy away from OPHS implementation. Another example was school policies that challenged the implementation of public health programs. As one manager described:

…we keep working with the school board to make sure that we can implement [the vaccine program]. Sometimes there are little barriers because we are on school grounds. For example, we must have parental consent and we know that there is no age of consent.

Municipal (local) economics were discussed as factors in policy implementation. **Municipal fiscal issues impacted the budgets of local public health units**. The province funds 75 % of public health while the municipality funds the remaining 25 %. These structures impacted funding for public health human resources in various ways. When municipalities implemented austerity measures such as hiring freezes and cuts in programs they were also implemented in some public health units negatively impacting staff capacity and thus OPHS implementation. For example, the fiscal austerity climate:

prevents [our casual staff] from becoming permanent. [] … it poses a barrier then to actually implementing the Standards because you don’t have the staff capacity to do that. And the wrinkle in our [region] is even though there’s a 75–25 funding split, if the [region] wants to cut you know a couple of million dollars they’re willing to lose …the leveraging from the province. So in many ways you’re losing three times that because of the leveraging.

#### Provincial level factors

There were five notable external provincial level factors that facilitated implementation.

The first is related to **Ministry-based consequences for not meeting the Standards and accountability**. Respondents noted that that the Ministry had been “extremely mute” about any repercussions related to not meeting the Standards. As a result, there was a perception that **no Ministry-based consequences** existed for failing to implement the policy. Participants identified likely consequences of not meeting the Standards as: a claw back of funding, funding withdrawal, or a fine. One participant felt that with the absence of penalties “the government does lose a little bit of credibility” with the Boards of Health. A STIP manager commented that the lack of penalties underscored a feeling that health units were unsupported by the province: “…the running joke is that the province doesn’t support health units. It doesn’t provide any real guidance to us. […] and all health units are on their own and what not.” Despite this uncertainty, there was **awareness of accountability agreements** between the Ministry and health units that were going to be created for monitoring purposes, but not be tied to funding. One respondent guessed that scoring low on an indicator would lead to “a meeting to discuss why [it was] not [reached]” as is done with others in the health sector. Respondents suggested that the Ministry would be open to documentation and rationales such as the community’s particular health needs or the size of the health unit to justify variances with meeting the Standards’ expectations. One respondent mentioned that the Ministry “has been very clear though that they will work with health units to address any type of shortfall.” Some participants said that not meeting a standard would imply the need for additional funding or raise the profile of a particular issue.

The second is the **supportive role of various provincial-level government departments or related agencies**. Public Health Ontario and the AIDS Bureau are examples:

I’m not sure health units would’ve done the research and picked up and implemented [AIDS programming such as anonymous and point-of-care testing], and bought the supplies for point of care without the AIDS Bureau leading that kind of charge.

The third provincial factor includes **Ministry activities that supported health units in building capacity to implement the policy**. For example, the Ministry provided communications to health unit leaders (managers) related to implementation of the OPHS through regional meetings across the province as well as teleconferences. This illustrates the inter-relationship that exists between provincial and organizational factors. On-going communication efforts have also been made through updates posted on a website. A participant reported that the website provided not only the OPHS 2008 and protocols, but also included background information, logic models, and other resources. Public health staff could also approach the province for support. As one STIP manager noted:

… If we would have a question about a specific item under the OPHS, we would go to the province. It happens very occasionally that we will go the province for stuff; for case management issues, if it’s an interpretation of a case definition […]

The fourth noteworthy factor supporting implementation is the **existence of other provincial policies that influence public health work**. Three examples of provincial policy that affect local implementation of the OPHS are: the School Food and Beverage Policy, Smoke-Free Ontario strategy and legislation, and the Ministry of Education’s Daily Physical Activity in Elementary Schools policy. These policies also drove public health activities and were seen as being in alignment with OPHS directions.

The fifth factor influencing implementation was **imbalances of provincial funding across programs**. Participants discussed that certain federal or province-wide programs such as Smoke Free Ontario received targeted or additional funding while other programs relied solely on their public health unit budgets. This meant that certain programs were better resourced than others which influenced OPHS implementation.

We are so small and we don’t have a lot of resources and because we are getting funded provincially for [tobacco] programming. I mean it sometimes seems unbalanced to the other programs. Why do they get so much training? Why couldn’t they go to conferences? How come they have all the swag?....So, it can seem very unbalanced.

This imbalance was frustrating for staff and managers who had to do more with less.

#### National or Federal Level Factors

While there are national or federal level influences on implementation of the policy interventions, these appear to be much less prominent than local/regional or provincial factors. **Resources available through national agencies** such as the Public Health Agency of Canada’s e-portal were one notable factor. Public health units report following the National Advisory Committee on Immunization (NACI) guidelines related to immunizing for hepatitis B and hepatitis A, and the Canadian STI guidelines, which helped them implement the OPHS.

#### Multilevel factors

Although most influences on OPHS implementation were identified as occurring at the local/regional level, provincial and national level influences were also felt. Further, some influences were related to more than one level. For example, **H1N1 influenza** was a local, provincial, and national public health priority in 2009 with resources and priorities at each level that influenced public health activities. Other examples include **multi-level initiatives** such as Canada Prenatal Nutrition Program, Canadian Physical Activity Guidelines; and various promotional events and weeks, such as National Non-Smoking Week and National Nutrition Month. Other initiatives were noted that originated from various branches of the Federal Government, such as First Nations and Inuit Health Branch.

## Discussion

This study helps to fill a gap that exists in the public health policy implementation literature. It showed that multiple levels of influence exist that impact the implementation of the public health policies. In addition, complex relationships exist among factors across and between levels. This is illustrated in Fig. 3 by dotted lines between levels. The factors aligned very closely with many of the components in Greenhalgh and colleagues’ Model of Diffusion in Service Organizations [23], which is used to guide the discussion. It is important to note that the Greenhalgh et al model was created as a composite framework based on a meta-narrative systematic review of the diffusion of innovations literature. Therefore, it is reasonable to assume that each element in the model is not relevant in every situation nor is it reasonable to expect that it would show up in every instance. Our data did not demonstrate relevance of some aspects of the model (e.g., aspects of the innovation itself such as trialability, capacity to evaluate the innovation), indicating that some influences were more important than others in this situation. Participants identified those that were most relevant and salient to them basing their interpretations of their own experiences. These interpretations might be different from others in similar or different situations. However, in our constant comparative analysis, each concept ‘earned’ its way into the results by appearing repeatedly in the data. At the same time, the factors influencing diffusion of innovation (which includes an implementation stage) in Greenhalgh’s framework were empirically grounded in a large body of literature.

Factors that were most clearly linked to the OPHS policy implementation and components in the model and demonstrating a powerful influence on implementation will be highlighted. These include: *attributes of the innovation itself*; *adoption by individuals; diffusion and dissemination;* the *outer context - interorganizational networks and collaboration; the inner setting - implementation processes and routinization; and, linkage at the design and implementation stage.* We discuss each of these components followed by implications for practice and policy, and study strengths and limitations.

### Model of Diffusion in Service Organizations and Implementation of a Policy Intervention in Public Health

#### Attributes of the innovation itself

*Attributes of the innovation itself* – that is, the OPHS policy - is a component in the model that influences uptake. End users of the innovation need to perceive relative advantage of the innovation for successful implementation [23, 30]. Many staff members identified that the policy was useful to justify programs, especially politically sensitive ones such as harm reduction. The fact that the OPHS are legislatively mandated is an attribute that could be used to leverage local implementation and could be helpful in securing resources that might otherwise be diverted to other municipal programs. Opportunity for reinvention of the innovation is another aspect of an innovation’s attributes that can support uptake [23]. Participants had mixed opinions in relation to the prescriptiveness of the new policy. Most participants felt that the policy was less prescriptive compared to the OPHS predecessor (the MHPSG), particularly in relation to the CDP program. The ability to tailor programs to meet local community needs was seen as a great advantage in the new policy - an opportunity for reinvention. On the other hand, staff involved in STIP programs did not see much change of the new policy with respect to prescriptiveness, since they have always had strict guidelines to follow for STI management including contact tracing. Similarly, the Bax, de Jong and Kooppenjan study of implementation of road policy in the Netherlands [31] noted that a policy implementation gap was the absence of precise policy objectives which allowed for discretionary power to deviate from the intended policy directions. They also argued that an implementation strategy needs to match the policy and implementation context. Another important lesson was that “local knowledge is indispensable to adapt a uniform package of measures to specific conditions” (p.880). In other words, implementation requires a certain amount of discretionary freedom to permit local tailoring. On the other hand, Bax and colleagues also reported that a lack of precision in their policy had risks characterised as: “‘not knowing how to’ (lack of proper information and communication), ‘not being able to’ (lack of competence and capacity), and ‘not wanting to’ (resistance)” ([31] p.873). This finding mirrors results of our parallel public health policy implementation paper which reported a key theme as, “you’ve told me what, now tell me how” [6]. A balance is needed that allows for tailoring of policies to local contexts along with the provision of resources and supports to facilitate implementation.

#### Adoption by individuals

In relation to the component *adoption by individuals*, Greenhalgh and colleagues [23] argue that being engaged in discourse around the innovation can help increase the meaning that is attached to the innovation. This has been found to influence uptake by individuals [23]. Further, when individuals’ ascribed meaning of the innovation matches how senior management and other stakeholders understand it, this can result in better uptake [22, 23, 32, 33]. It is likely that staff involvement in policy development as well as active discussions around it during implementation, enhanced the meaning attached to the policy. It also increased its perceived advantage. A longitudinal case study involving two Swedish national evidence-based policies aimed at improving health and social care indicated that active involvement and dialogue of professional organizations (stakeholders) during policy development allowed for a quick implementation start up, and improved the chances for a positive response from the target group [34]. Normalization process theory also highlights the importance of individuals’ understanding the meaning of the innovation, known as coherence [32, 33]. The authors argue that this can influence implementation and uptake of practice innovations and was demonstrated in our analysis through participants’ critical reflection on the policy’s implications for practice and communities.

Greenhalgh et al. [23] also drew on strong indirect evidence from work by Hall and Hord [35]. They showed that if participants understand the consequences of the intervention, they will more likely adopt it. In contrast, participants in our study were unclear about the consequences of not meeting the policy standards. Many felt there were none or that consequences would be tied to funding. Despite this lack of clarity, many were aware that accountability agreements were soon to be released that would clarify expectations related to meeting the standards. This points to a relationship between factors in the outer setting (i.e., accountability and consequences) and adoption by individuals. Similarly, in a Swedish case study of a national public health policy implemented in two municipalities, Jansson, Fosse and Tillgren [36] reported that that the policy lacked relevance to their context, was not targeted to local needs, and lacked specific requirements. Engaging staff in discussions and communicating information related to consequences surrounding public policy during implementation appears to be important to improve uptake.

#### Diffusion and dissemination

*Diffusion and dissemination* is another component in the model which has been shown to influence implementation [23]. As noted earlier, a number of public health service providers were unaware of the new policy despite senior and middle managers’ attempts to communicate them to staff. This was reported to a greater and lesser extent depending on the health unit. Many providers felt that policy implementation was a management responsibility, while they were charged with service delivery. This meant that the policy held little meaning in their workload for many front line staff, which influenced their degree of interest in learning about it. It is not surprising then that most managers and service providers who were either engaged with Ministry staff in developing the policy or provided feedback during its development felt particularly engaged and positive about their experiences. Others have found that government policy implementation at the municipal level was successful when it was desired and understood and there were resources provided to support implementation [36].

#### The outer context: interorganizational networks and collaboration

The *outer context* is another critically important consideration in the model [23]. For example, the structure and quality of social networks were identified as a powerful influences on adoption of innovations [23]. Health unit staff often described the value of working with other regional and provincial networks to support their public health work. In this study, horizontal networks of peers were found to be supportive for program implementation, in particular for tobacco programs, as a positive “intentional spread strategy” [23] p. 609. Similarly, Greenhalgh et al. [23] noted that informal interorganizational networks such as homophilous networks or networked provider agencies that share a common management and governance structure can influence implementation. Related to the value of networks, a notable area that was identified in our results was the influence of external community partnerships with other sectors, such as primary care and schools. Input from and influence of these community partners was a significant factor influencing implementation of the OPHS. This is likely related to the value placed on collaborations and partnerships as a foundational principle in the OPHS [5].

Himmelman argues that collaborations demand much more in terms of shared resources; degree of trust; commitment to a common goal; and sharing of risks, responsibilities and rewards, than do networks [37]. Greenhalgh et al considered collaborations in their review, but noted that they were focused on rapid cycle quality improvement projects rather than the implementation of innovations [22]. Annor and colleagues [38] argue that how individuals and organizations interact with each other should be a major focus in policy implementation. Since a single organization cannot solve complex problems alone, working in collaboration is essential for success. They report on a case study of public mental health policy implementation in England involving local implementation teams to deliver the policy. They reported several challenges in working in collaborations including: a lack of common understanding of key definitions (i.e., mental health), funding projects with existing resources, low priority placed by partners on public mental health initiatives, and the presence of a dominant partner that controlled resources and held power which led to competition between stakeholders.

A qualitative study of implementation of the Ontario School Food and Beverage Policy showed that schools’ external partnerships with food suppliers and food industry were critical for successful policy implementation [39]. Strong collaborations with external partners appears to be essential for successful implementation of public health policies. The outer context also takes into account the social-political climate. Although the policy push from the Ministry was felt by those in management, it had less impact on health service providers. However, all participants noted that local politics including municipal budgets and directions from the Board of Health impacted implementation and had an influence on programming decisions. This is not surprising given the funding structure of the majority of public health services in ON is shared between the province (75 %) and the municipality (25 %).

#### The inner setting: implementation processes and routinization, and system readiness for innovation

The *implementation processes and routinization* component of the model involves having senior and middle management and leadership support and commitment to the innovation to influence uptake [23]. Leadership is also noted as an important system antecedent in the model which can help influence receptivity for change in the organization [23]. Alignment with the goals of leaders and the innovation is also important but the evidence for this association is not as strong. Others identify that having leaders set priorities and manage the process of implementation is important [30, 36]. In this study, the role of leaders in policy implementation was critical not only in providing direction for staff, but also in supporting their implementation efforts. Staff had varying opinions on the style of leadership and management approaches. How management styles influenced policy implementation was not clear, however, and deserves more exploration. Medical Officers of Health were seen as having great influence on program and services directions. Not surprisingly, a lack of leadership (a vacancy in senior leadership) or local municipal pressures were seen as a barrier to implementation. This tension was seen in some health units more than others. Participants expected more ‘top down’ direction and support from their leaders in these health units.

Our study showed that policy implementation was heavily integrated into health unit planning processes. Operational planning and strategic planning documents often referenced the OPHS. Greenhalgh’s model [22, 23] does not discuss the influence of planning processes in terms of implementation. However, the process of implementation, including the constructs of planning, engaging, executing, reflecting and evaluating was identified by Damschroder and colleagues [40] as a key domain in their consolidated framework for implementing research into practice. Planning processes were highly apparent influencing implementation in most health units. Planning documents were tied to financial costs and public health human resource allocation. Others have reported value in participating in joint planning processes such as the creation of community health profiles and policy dialogues to support implementation of integrated community plans [41].

Greenhalgh and colleagues’ model does raise the issue of having dedicated and adequate time and resources to implement the innovation as a component of *system readiness for innovation* [22, 23]. However, adding planning processes as a factor into the model may be warranted especially for highly bureaucratic organizations in which such processes are the norm.

#### Linkage at the design and implementation stage

The model discusses the importance of *linkage at the design and implementation stage* with developers and potential end users of the innovation. Engagement is critical to ensure that everyone’s perspectives are considered [23]. The study revealed that staff viewed their involvement in the development of the Ministry policies very positively. Further the Ministry’s support and constant feedback through webinars and road shows during the roll out of the policy was greatly appreciated by many participants. The model indicates that support from the change agency, e.g., government, in the form of ongoing dialogue and networking among organizations to support the change can be valuable for uptake as well as the provision of “augmented product” support (p. 598) such as a help desk [23].

### Implications for future provincial-level public health policy implementation

Our analysis demonstrated an uneven level of awareness of the OPHS and related documents at the time of roll out. In addition, there was an uneven level of engagement with the policies at the organizational level. Some leaders took an approach that satisfied their obligations for management accountability, e.g., they held staff meetings to discuss the Standards or they retrospectively ensured that their operational plans addressed them. Others, perhaps on account of their personal leadership style, worked in a pro-active manner by incorporating the Standards into organizational strategic plans or by creating a culture where staff felt supported in their implementation of the policies. Langley and Denis [21] suggest that traditional approaches to implementation, as we’ve presented here using the Greenhalgh et al., model [22], and perhaps as the Ministry envisioned, can be enhanced with attention to context-specific factors. The authors suggest three focal points: 1) attention to the uneven benefits that might be realized by different stakeholders (“what’s in it for whom”), 2) attention to values held by different groups, 3) understanding what is essential (in the policy) and what is in the soft periphery that can be adjusted to the context. A change management strategy could be included in a transition/implementation plan in the next set of standards [42] using a lens that positions health units as political systems rather than rational systems [21].

Our study strongly draws attention to the importance of ensuring policy cohesiveness to support successful implementation. Some policies are easier to implement than others as they might differ with respect to the degree of change required, resource requirements, complexity of the policy setting, etc. Sometimes the policy itself might have somewhat conflicting objectives, as was the case in our study, making it difficult to implement as intended. Repeatedly we heard that the policy was intended to be responsive to local community needs. Further, the policy explicitly supported collaboration with community agencies (one of the key principles) to promote health and prevent illness, and respondents noted that their community partners sometimes had particular requests from them. These collaborative directions are in line with traditional public health activity. However, the policy also took a stronger evidence-based approach than the previous MHPSG; in other words, health units were asked to carry out reviews of the literature to understand problems and develop programs with demonstrated effectiveness. This direction is in line with the general trend in the larger health sector and in public health to provide health services that are grounded in evidence [31, 34, 43, 44]. Thus both of these objectives – local responsiveness and research-based programming – are reasonable in their own right. Taken together, however, many respondents did indeed speak to a practice tension that arose when trying to implement the overall policy related to conflicting values of different forms of evidence. For example, a local demand or problem was not resolved because there was a lack of research literature (a form of evidence) to support an effective solution, or the literature suggested an alternative solution from what partner agencies demanded (community need, which is another form of evidence). Participants clearly felt uncomfortable when local needs could not be met because of a lack of research evidence. In addition, discomfort was felt when local needs were being met, but initiatives fell outside of the scope of the OPHS. An implication for future policy implementation is to ensure greater policy cohesiveness or mechanisms about how to manage these practice conflicts related to the policy.

### Limitations and strengths

There are a few limitations that should be noted in reference to this work. We involved six health units that we identified based on their different contexts; however, they may not be representative of all health units in Ontario. In addition, we were not able to engage all health unit staff members equally. For example epidemiologists and planners were underrepresented and these staff would likely have been quite engaged in policy implementation decisions. We did, however, obtain valuable input from public health staff at all levels of authority from service providers to senior leaders. Further, we did not seek input from members of Boards of Health who could have provided unique perspectives on implementation and the policies themselves. We selected CDP and STIP programs as exemplar programs in order to keep the study manageable, to provide a useful comparator and to enhance our ability to track changes over a five year period within a similar cohort. This decision did result in limited knowledge of the influence of the OPHS in other program areas. Despite these limitations, we have obtained rich input from a wide variety of stakeholders in public health including services providers who typically are not asked for their impressions on policy, and are ultimately the ones who provide the programs and services to meet the OPHS. Another strength of this research is that it reflects an integrated knowledge translation approach, where practitioners and policymakers were co-investigators on the research team. Some of them contributed to the writing of this paper. Their additional insights about the data served to bring credibility to our collective interpretation.

## Conclusions

This study explored processes and factors influencing implementation of a new provincial public health policy in Ontario, Canada. Although many health unit service providers were unaware of the policy change, engagement in developing the policy or provision of feedback by health unit staff at the front line as well as the management level was positively received. Implementation of the policy was most evident in planning processes that incorporated many internal stakeholders as well as community partners. The policy was also incorporated in operational planning documents such as logic models. Factors influencing implementation were closely aligned to components of Greenhalgh and colleagues’ Diffusion of Innovations in Service Organizations framework. Most notably, the nature of the policy itself (e.g., its prescriptiveness), organizational leadership, planning processes, governance, health human resources, local and regional economics, as well as provincial and national factors such as other policies, best practice evidence and networks had an influence on implementation. Our results provide empirical support for components of Greenhalgh et als’ framework [23] and suggest that two additional components be considered – the role of external partnerships with other sectors and the influence of planning processes. The constructs – planning processes and cosmopolitanism (links to external organizations) – are identified in the more recent Consolidated Framework for Implementation Research [40]. These influences are important to consider by public health organizations when implementing new or revised provincial public health policies given that they constantly change and evolve over time. The RePHS team will continue to explore changes in policy implementation and impact over time in ON as well as BC.

## Additional files

Additional file 1: History of the Ontario Public Health Standards Development. This files contains a description of the history of the development of the standards as well as an image to illustrate the stages. (DOCX 43 kb) Additional file 2: Focus Group and Interview Guides. This file contains all of the focus group and interview guides used for all participants. (DOCX 27 kb)

## Abbreviations

BC: British Columbia
CDP: chronic disease prevention
HPPA: Health Protection and Promotion Act
MHPSG: Mandatory Health Programs and Services Guidelines
MOHLTC: Ontario Ministry of Health and Long-Term Care
ON: Ontario
OPHS: Ontario Public Health Standards 2008
RePHS: Renewing Public Health Systems
STIP: sexually-transmitted infection
