# Supplementary material for: Moving towards a new vision: implementation of a public health policy intervention
Source: BMC Public Health. 2016 May 17;16:412. doi: 10.1186/s12889-016-3056-3 (PMC4869271; doi:10.1186/s12889-016-3056-3)
Supplement: Additional file 1: — History of the Ontario Public Health Standards Development. This files contains a description of the history of the development of the standards as well as an image to illustrate the stages. (DOCX 43 kb) [file 12889_2016_3056_MOESM1_ESM.docx]

Additional file 1

**History of the Ontario Public Health Standards Development**

Five years after the 2003 SARS outbreak, the Ontario Public Health Standards (OPHS 2008) [1] were released. Figure 1 illustrates the history of the process of initiation, development, and roll out of the policies up to 2010 when this program of research was initiated.

In 2006, a comprehensive process to review the 1997 legislated public health minimal requirements [Mandatory Health Programs and Services Guidelines (MHPSG)] was initiated [2]. Provincially the review was sparked as a result of numerous reports following the 2003 SARS outbreak [3, 4], including the Ministry’s action plan – *Operation Health Protection: An Action Plan to Prevent Threats to our Health and to Promote a Healthy Ontario* [5]; and the final report from the Capacity Review Committee in 2006, *Revitalizing Ontario’s Public Health Capacity* [6]. This latter report recommended that the MHPSG [2] be replaced with program standards to be continually revised in an ever-greening process.

Led by the Ontario Ministry of Health and Long-Term Care (MOHLTC) in collaboration with the former Ministry of Health Promotion and the Ministry of Children and Youth Services, a Program Standards Technical Review Committee guided the review process and oversaw the development of the renewed public health standards starting in 2006. Standards were expected to fit within public health’s fiscal envelope or public health funding. There was to be increased emphasis on accountability, coordination and collaboration across the health system, as well as financial sustainability. The Ontario Public Health Standards (OPHS) development included stakeholder engagement such as, a Reference Panel from key organizations and professional bodies, an Inter-Ministry Committee, all boards of health, and close to 90 other public health stakeholders.

The draft OPHS were submitted to the Chief Medical Officer of Health in April, 2007 with an expectation that “a robust and comprehensive roll-out strategy” would be included with training supports. These were approved in principle by all three ministries in June 2007. On the Technical Review Committee’s advice, government continued to develop protocols to support specific requirements within the OPHS to achieve greater standardization in province-wide implementation. This was aligned with 2007 amendments to the *Health Protection and Promotion Act* (HPPA) [8], which allow for the incorporation of documents such as protocols into the OPHS and their rolling incorporation to facilitate revision. In 2007, Protocol Development Teams began drafting protocols, such as a Population Health Assessment and Surveillance Protocol and Infectious Diseases protocol [9, 11]. Numerous stakeholders had an opportunity to comment on drafts in 2008. Guidance documents were also developed, updated or referenced. The OPHS and 26 protocols were released in October 2008 and came into effect in January 2009. They were published by the Ontario Ministry of Health and Long-Term Care, pursuant to Section 7 of the HPPA. The OPHS continue to be updated, with the most recent revisions to the 2008 Standards published in 2014 [17] with an additional protocol. In support of the OPHS release, the former Ministry of Health Promotion and Sport released nine guidance documents in 2010. Recruitment to the study began at this time.

**Figure 1:** History of the Development and Implementation of Public Health Policy in Ontario up to 2010 (Initiation of the Study)

1. Ontario Ministry of Health and Long-Term Care. Ontario Public Health Standards, 2008. Toronto: Queen's Printer for Ontario; 2008.

2. Ontario Ministry of Health and Long-Term Care. Mandatory Health Programs and Services Guidelines Toronto: Queen's Printer of Ontario; 1997.

3. Ontario Expert Panel on SARS on Infectious Disease Control. For the public's health: A plan of action. Final report of the Ontario Expert Panel on SARS and Infectious Disease Control.2004.

4. National Advisory Committee on SARS and Public Health. Learning from SARS: Renewal of public health in Canada. 2003.

5. Ontario Ministry of Health and Long-Term Care. Operation Health Protection: An action plan to prevent threats to our health and to promote a healthy Ontario. 2004.

6. Tamblyn S, Hyndman B, Bewick D, Chow L, Hicks T, Munter A et al. Revitalizing Ontario's public health capacity: The final report of the capacity review committee.2006.

7. Ministry of Health and Long-Term Care. The Ontario Public Health Standards (OPHS) and Protocols, 2008. Service Ontario; 2014.

8. Ministry of the Attorney General: Government of Ontario. Health Protection and Promotion Act,R.S.O. 1990, Chapter H.7. Service Ontario eLaws 2011.

9. Ontario Ministry of Health and Long-Term Care. Ontario Public Health Standards 2008: Population health assessment and surveillance protocol.2008.

10. Ontario Public Health Standards: Ontario Ministry of Health and LongTerm Care. Tanning Beds Compliance Protocol. Toronto: Ontario Ministry of Health and Long-Term Care2014.

11. Ontario Ministry of Health and Long-Term Care. Ontario Public Health Standards 2008: Infectious Diseases Protocol.2009.

12. Ontario Ministry of Health and Long-Term Care. Ontario Public Health Standards 2008. Toronto: Queen's Printer for Ontario; 2014.

13. Kothari A, Gore D, MacDonald M, Bursey G, Allan D, Scarr J. Chronic disease prevention policy in British Columbia and Ontario in light of public health renewal: a comparative policy analysis. BMC public health. 2013;13(1):934.

14. Pinto AD, Manson H, Pauly B, Thanos J, Parks A, Cox A. Equity in public health standards: a qualitative document analysis of policies from two Canadian provinces. IntJ EquityHealth. 2012;11:28. doi:1475-9276-11-28 [pii];10.1186/1475-9276-11-28 [doi].

15. Regan S, MacDonald M, Allan DE, Martin C, Peroff-Johnston N. Public health human resources: a comparative analysis of policy documents in two Canadian provinces. Human resources for health. 2014;12(1):13.

16. Valaitis R. Primary Care and Public Health Collaboration: The Time is Ripe. Trillium: Primary Health Care Research Day June 19, 2013; Toronto2013.
