# Supplementary material for: Moving towards a new vision: implementation of a public health policy intervention
Source: BMC Public Health. 2016 May 17;16:412. doi: 10.1186/s12889-016-3056-3 (PMC4869271; doi:10.1186/s12889-016-3056-3)
Supplement: Additional file 2: — Focus Group and Interview Guides. This file contains all of the focus group and interview guides used for all participants. (DOCX 27 kb) [file 12889_2016_3056_MOESM2_ESM.docx]

**Additional File 2**

**Focus Group and Interview Guides**

**ON Public Health Frontline Staff Focus Group Guide**

READ BEFORE BEGIN INTRODUCTION:

Before we begin, we would like to mention that some of these questions may seem more challenging than others. There is no requirement for each of you to answer all of the questions and your answers are not being judged. Please feel free to respond only to the questions that you feel comfortable answering.

It will be helpful for the transcriptionist if you could state your name each time you answer a question or add to the discussion.

Before we get into the questions, it will be helpful for us to get some demographic background information about each of you. Can we please go around the table and state your name, job title/role, program, and length of time in current position, and discipline.

**Public Health Frontline Staff**

**Introduction**

**(for the interviewer to set the context)**

We are interested in learning about the implementation of the 2008 Ontario Public Health Standards (OPHS) in your Health Unit. Since the OPHS is a policy intervention, when we refer to “policies”, we are referring to the OPHS 2008 document and the associated legislated protocols.

Further, we are interested in learning more about the impact of the implementation of the new policies on two programs - Chronic Disease Prevention (CDP) and Sexually Transmitted Infection Prevention and Control (STIP).

For CDP Groups

We are aware that CDP typically involves many topics. Therefore, to keep the scope of the research manageable we have refined aspects of CDP programming that we are interested in, including: healthy eating, physical activity, and tobacco.

For STIP Groups

We are aware that STIP typically involves many topics. Therefore, to keep the scope of the research manageable we have refined aspects of STIP that we are interested in, including: screening, education, immunization, harm reduction, treatment, and contact tracing/partner notification.

**Focus Group Questions**

**Background**

- Briefly describe your role within the HU and specifically your involvement with the CDP/STIP program activities/initiatives.
- What have you heard about the policies and from where?

**Change in activities over time**

1. Did you ever have discussions/presentations about the CDP/STIP changes from the prior Mandatory Health Programs and Services Guidelines?
   1. If yes, please describe.
   2. What did you think of the discussion of changes?
   3. What supports or activities (e.g., team discussions) are ongoing to discuss these changes?
2. How has your role changed with the implementation of the OPHS?
3. What do you think about the change?
4. How do you think your colleagues think and feel about any changes to the CDP/STIP activities they are involved in, which have resulted from the implementation of the OPHS?

**Evidence**

1. In general, what informs or guides your practice

(Prompts: evidence, theory, literature, observing/talking to peers/experts, professional practice guidelines)?

1. What does the word evidence mean to you?

(Prompts: policy, by-law)

- 1. What constitutes evidence for you? (Could ask this if it isn’t elicited by the first part of the question)

1. What evidence/strategies do you use to guide/inform your practice as they/it relate(s) to the OPHS?
2. What kinds of mechanisms are in place for you to foster the use of evidence if any?

(Prompts: in-services/workshops, continuing education, looking at literature)

1. How do you think evidence is used in relation to the CDP/STIP activities?
2. Do you encounter barriers regarding implementing evidence in your practice?

(Prompts: for what the barriers are if needed)

**Leadership**

1. How does your work environment support you in terms of your responsibilities within the new policies?

(Prompts: role of manager/directors, other)

1. Do you have an opportunity to provide feedback or input on the CDP/STIP implementation/planning/evaluation of programming?

**ON Managers Focus Group and Interview Guide**

READ BEFORE BEGIN INTRODUCTION:

Before we begin, we would like to mention that some of these questions may seem more challenging than others. There is no requirement for each of you to answer all of the questions and your answers are not being judged. Please feel free to respond only to the questions that you feel comfortable answering.

It will be helpful for the transcriptionist if you could state your name each time you answer a question or add to the discussion.

Before we get into the questions, it will be helpful for us to get some demographic background information about each of you. Can we please go around the table and state your name, job title/role, program, length of time in current position, and discipline.

**Managers**

**Introduction**

**(for the interviewer to set the context)**

We are interested in learning about the implementation of the 2008 Ontario Public Health Standards (OPHS) in your Health Unit. Since the OPHS is a policy intervention, when we refer to “policies”, we are referring to the OPHS 2008 document and the associated legislated protocols.

Further, we are interested in learning more about the impact of the implementation of the new policies on two programs - Chronic Disease Prevention (CDP) and Sexually Transmitted Infection Prevention and Control (STIP).

For CDP Groups

We are aware that CDP typically involves many topics. Therefore, to keep the scope of the research manageable we have refined aspects of CDP programming that we are interested in, including: healthy eating, physical activity, and tobacco.

For STIP Groups

We are aware that STIP typically involves many topics. Therefore, to keep the scope of the research manageable we have refined aspects of STIP that we are interested in, including: screening, education, immunization, harm reduction, treatment, and contact tracing/partner notification.

**Focus Group / Interview Questions**

**Background**

1. Briefly describe your role within the HU and specifically your involvement with the CDP/STIP program activities/initiatives.

**Change in activities over time**

1. How has your role changed with the implementation of the OPHS?
2. What do you think about the change?
3. How do you think your colleagues think and feel about any changes to the CDP/STIP activities they are involved in, which have resulted from the implementation of the OPHS?
4. How have the following changed since the implementation of these CDP/STIP activities:
   1. planning,
   2. implementation,
   3. and evaluation of programs?

**Evidence**

1. In general, what informs or guides your practice?

(Prompts: evidence, theory, literature, observing/talking to peers/experts)

- 1. What has the most influence in guiding your practice?

1. What does the word evidence mean to you?

(Prompts: policy, by-law, want to capture in this question the use of evidence regarding decision-making in program planning)

- 1. What constitutes evidence for you? (Could ask this if it isn’t elicited by the first part of the question)

1. What evidence or information was used to inform the development of the CDP/STIP program activities as they relate to the OPHS?
2. What is the process for applying evidence in program development?
   1. How are the OPHS, protocols, and guidance documents used?
   2. At what level(s) are decisions made in terms of what evidence is used?

(Prompt: Who decides what evidence is used?)

1. What influences how and what evidence is used?

(Prompts: science, research, social/cultural/political environment)

1. Do you have an opinion on their use?
2. Has there been an effort to create/develop provincial evidence as a result of public health renewal?
3. Are there barriers to implementing evidence?

**Planning**

1. Describe the planning process for developing any guidance documents or other relevant documents to guide programs/services.

(Prompts: who, when, how often, silo vs. integrated).

1. What do you think about the planning process?

(Prompts: collaborative, inclusive, effective, useful)

1. How are communities involved in decisions about, and CDP/STIP activities?

**Leadership**

1. How were the OPHS communicated to staff?
2. What general processes, including those specific to decision-making, influenced the implementation of the new policies?
3. What other factors influence decision-making around implementation?

(Prompts: evidence)

**Implementation**

1. Describe the implementation process of/for the OPHS in the CDP/STIP program area in your Health Unit.

(Prompts: who, what, when, how, etc.)

1. What bodies/tables/venues have been established for Health Units, Ontario Ministries, and Agencies, e.g., MOHLTC, Ministry of Health Promotion, Ontario Agency for Health Protection and Promotion, and other stakeholders to interact to support implementation?

(Prompts: evidence, data, direction, lessons learned)

1. What, if any, do you believe would be the consequences from government of any failure to implement the OPHS?
2. What are the provincial initiatives/strategies influencing the implementation of CDP/STIP policies?
3. How, if at all, are these CDP/STIP activities aligned with other local, regional, provincial, and national initiatives?
4. How has collaboration enabled the implementation of CDP/STIP policies?

(Prompts: with whom, with what effect)

1. How does the particular governance model for your Health Unit influence the implementation process or the outcomes of CDP/STIP policies?
2. How is your Health Unit dealing with economic changes in relation to the implementation of the OPHS?
3. Since the OPHS are relatively new, not everyone may be aware of them. What did you know about the OPHS prior to your recruitment to this study and how has your knowledge changed about them?

**Evaluation**

1. How is your Health Unit monitoring performance on the OPHS policies?
2. Who (what position within your Health Unit) is developing the mechanisms to monitor/evaluate performance?
3. What are the accountability mechanisms in your Health Unit related to OPHS in relation to CDP/STIP policies?
4. Are there consequences in terms of accountability?
5. How well do the performance monitoring processes work in achieving implementation?
6. How do you define successful implementation of the OPHS policies?
7. How does your Health Unit define successful implementation?

**Partnerships external to the Health Unit**

1. What new partnerships have been formed and how have old partnerships changed since the implementation of the OPHS?
   1. What is the purpose of these partnerships?
   2. How do these partnerships function?
   3. Who is involved in these partnerships?

**Senior Management**

**(Directors/ Medical Officers of Health/ Associate Medical Officers of Health)**

**Focus Group and Interview Guide**

**Introduction**

We are interested in learning about the implementation of the 2008 Ontario Public Health Standards (OPHS) in your Health Unit. Since the OPHS is a policy intervention, when we refer to “policies”, we are referring to the OPHS 2008 document and the associated legislated protocols.

Further, we are interested in learning more about the impact of the implementation of the new policies on two programs - Chronic Disease Prevention (CDP- including healthy eating, physical activity, and tobacco ) and Sexually Transmitted Infection Prevention (STIP including: screening, education, immunization, harm reduction, treatment, and contact tracing/partner notification).

Demographic Information

- Before we begin, please state your name, role/title, discipline, number of years in current role and number of years in public health.

**Interview Questions**

**Background**

1. Briefly describe your role within the Health Unit (HU).

**Change in activities over time**

1. How has your role changed with the implementation of the OPHS?
2. What do you think about the change?
3. How have the following changed since the implementation of these CDP/STIP activities:
   1. planning,
   2. implementation,
   3. and evaluation of programs?

**Leadership**

1. How were the OPHS communicated to staff?
2. What general processes, including those specific to decision-making, influenced the successful implementation of the new policies?
3. What other factors influence decision-making around implementation of the new policies?

**Implementation**

1. Describe the implementation process of/for the OPHS in the HU in general and in CDP/STIP programs.
2. What, if any, do you believe would be the consequences from government of any failure to implement the OPHS?
3. How does the particular governance model for your Health Unit influence the implementation process or the outcomes of CDP/STIP policies?

**Evaluation**

1. How is your Health Unit monitoring performance on the OPHS policies?
2. Who (what position within your Health Unit) is developing the mechanisms to monitor/evaluate performance?
3. What are the accountability mechanisms in your Health Unit related to OPHS in relation to CDP/STIP policies?
4. Are there consequences in terms of accountability?
5. How well do the performance monitoring processes work in achieving implementation?
6. How does your Health Unit define successful implementation of the policies?

**Equity**

1. Has consideration of health equity been part of the implementation of the OPHS specific to CDP/STIP activities?
   1. If yes, how has this been achieved? If no, why not?
2. When health inequities in relation to CDP/STIP are identified, how does your HU respond?

**Public Health Human Resources**

1. How has implementation of the OPHS influenced staffing in your health unit, specific to CDP/STIP?
2. Do you have what you need in terms of resources and appropriate staffing with the right skill level to carry out the changes that have occurred as a result of the implementation of the OPHS specific to CDP/STIP?
3. Have new roles been created/ implemented or have current roles changed as a result of the OPHS?
4. Are there new competencies (knowledge, skill, and judgment) required to effectively meet the OPHS? If so, what are they?
5. Are there organizational activities to support staff to apply and/or gain the required competencies? If so, what are they?

**Workforce Planning – For senior leadership with HHR responsibility**

1. Does your health unit have a public health human resources (PHHR) plan/strategy?
2. How often does PHHR planning occur?

*If we could obtain a copy of the workforce plan that would be helpful for the analysis.*

**Collaboration**

1. Do you think the OPHS influenced the way your health unit works with primary care in STIP/CDP in any way? If so, how?
2. Has implementation of the OPHS influenced how the CDP/STIP program works with other divisions/programs in your health unit?
